# Quantification and evolution of mitochondrial genome rearrangement in Amphibians

**DOI:** 10.1186/s12862-021-01755-3

**Published:** 2021-02-09

**Authors:** Jifeng Zhang, Guopen Miao, Shunjie Hu, Qi Sun, Hengwu Ding, Zhicheng Ji, Pen Guo, Shoubao Yan, Chengrun Wang, Xianzhao Kan, Liuwang Nie

**Affiliations:** 1 School of Biological Engineering, Huainan Normal University, Huainan, Anhui 232001 People’s Republic of China; 2 College of Life Science, Anhui Normal University, Wuhu, Anhui 241000 People’s Republic of China; 3 Anhui Key Laboratory of Low Temperature Co-Fired Materials, Huainan Normal University, Huainan, 232001 People’s Republic of China; 4 Key Laboratory of Industrial Dust Prevention and Control and Occupational Health and Safety, Ministry of Education, Huainan, 232001 People’s Republic of China; 5 Anhui Shanhe Pharmaceutical Excipients Co., Ltd., Huainan, 232001 People’s Republic of China; 6 Department of Biostatistics, Bloomberg School of Public Health, Johns Hopkins University, Baltimore, MD 21205 USA; 7 Life Science and Food Engineering College, Yibin University, Yibin, Sichuan 644000 People’s Republic of China

**Keywords:** Mitogenomics, Amphibians, qMGR, Rearrangement score, Rearrangement frequency, Phylogenetic characteristics

## Abstract

**Background:**

Rearrangement is an important topic in the research of amphibian mitochondrial genomes ("mitogenomes" hereafter), whose causes and mechanisms remain enigmatic. Globally examining mitogenome rearrangements and uncovering their characteristics can contribute to a better understanding of mitogenome evolution.

**Results:**

Here we systematically investigated mitogenome arrangements of 232 amphibians including four newly sequenced Dicroglossidae mitogenomes. The results showed that our new sequenced mitogenomes all possessed a *trnM* tandem duplication, which was not exclusive to Dicroglossidae. By merging the same arrangements, the mitogenomes of ~ 80% species belonged to the four major patterns, the major two of which were typical vertebrate arrangement and typical neobatrachian arrangement. Using qMGR for calculating rearrangement frequency (*RF*) (%), we found that the control region (CR) (*RF* = 45.04) and *trnL2* (RF = 38.79) were the two most frequently rearranged components. Forty-seven point eight percentage of amphibians possessed rearranged mitogenomes including all neobatrachians and their distribution was significantly clustered in the phylogenetic trees (*p* < 0.001). In addition, we argued that the typical neobatrachian arrangement may have appeared in the Late Jurassic according to possible occurrence time estimation.

**Conclusion:**

It was the first global census of amphibian mitogenome arrangements from the perspective of quantity statistics, which helped us to systematically understand the type, distribution, frequency and phylogenetic characteristics of these rearrangements.

## Background

As semi-autonomous organelles, mitochondria retain their own genomes and participate in many essential biological processes in eukaryotic cells such as energy transduction and intermediary metabolism. The content of vertebrate mitogenomes is conservative, including 13 protein-coding genes (PCGs), 22 tRNA genes (*trns*), 2 rRNA genes (*rrns*) and a control region (CR) with replication and gene transcriptional regulatory signals [1, 2]. Because of the moderate evolutionary rate of the mitogenome, it has been an excellent molecular marker for phylogenetic studies [3–6].

The gene order of vertebrate mitogenomes is as conservative as its content, such that human, mouse, clawed frog and zebrafish all share the same gene arrangement in their mitogenomes [7–10]. However, with an increasing number of different species’ mitogenomes completely sequenced, gene rearrangements have been reported in many different taxa. Generally, these rearrangements are inferred to the results from events such as gene transposition, gene inversion, gene duplication, and gene loss. Summaries and comparisons of animal mitogenome rearrangements have been reported [3, 11]. An initial important study undertaken by Boore summarized all gene arrangements known for Metazoa, based on no more than 100 mitogenomes available at that time [1]. Further comparative studies of mitogenome arrangement have subsequently been published (e.g., [12–15]). In addition, several possible rearrangement models were proposed to explain mechanisms, including tandem duplication–random loss (TDRL) [16, 17], tandem duplication and non-random loss (TDNL) [18, 19], intramitochondrial recombination [20, 21], etc. Using the CREx and TreeREx programs, based on four rearrangement models, other studies have tried to find common intermediate steps (common intervals) in two or more gene orders being investigated to elucidate the evolution of mitogenome rearrangements in different lineages such as insects and crabs [22–25].

In general, most animals retain the conserved rather than rearranged mitogenome components and order. The conservative arrangement is called the “typical vertebrate (mitogenome) arrangement” in vertebrates and the “typical invertebrate (mitogenome) arrangement” in invertebrates [1, 26]. To date, the number of vertebrate species with typical vertebrate arrangement accounts for more than half of all species for which mitogenomes have been determined. Among the amphibians, neobatrachians are the majority of frogs, accounting for ~ 92% of the total, over 6600 species (AmphibiaWeb, http://www.Amphibiaweb.org/, accessed February, 2020). Most sequenced neobatrachian frogs possess a derived mitogenome arrangement, “typical neobatrachian arrangement” [14, 27, 28]. Furthermore, there are some other types of rearrangements in addition to typical neobatrachian arrangement in amphibians. For example, the mitogenome of the neobtrachian frog *Limnonectes bannaensis* lacks *trnA*, *trnN*, *trnC* and *trnE* and contains a tandem duplication of *trnM* [29], the mitogenome of the caecilian *Crotaphatrema lamottei* includes the duplications of *trnF*, *trnP* and *trnT* and lack of *trnK* [30], and there are two CRs found in the mitogenomes of the neobatrachian frogs *Mantella madagascariensis* [31] and *Rhacophorus schlegelii* [32]. In addition, while *nad6* is located between *nad5* and *cob* genes within typical vertebrate mitogenome, it is rearranged to between *rrnL* and *nad1* in the mitogenome of the plethodontid salamander *Aneides hardii* [33]. Furthermore, in some amphibians intraspecific variation in mitogenome rearrangements has been reported [34]. These findings inspire us to further explore the landscape of mitogenome rearrangements in amphibians.

Except for earlier studies on a few species of amphibians, most studies have focused only on a few lineages rather than a more global and systematic analysis. Also, previous studies have paid little attention to the role of individual genes in rearrangement. Recently we proposed a method, qMGR, for quantifying mitogenome rearrangement and developed a web service (http://qmgr.hnnu.edu.cn/) that provides large-scale and accurate analysis of mitogenome rearrangement information [35].

In this study, we newly determined mitogenomes for four frogs in the neobatrachian frog family Dicroglossidae, and found that they all had a tandem duplication of *trnM* (IQMM *trn* cluster), which was not a feature exclusive to this family [36, 37]. To identify common characteristics of mitogenome rearrangement in amphibians, we then focused on the study of gene rearrangement patterns of all known amphibian mitogenomes, quantified the rearrangement frequency *(RF)* for each single gene and the rearrangement score *(RS)* for each mitogemome by qMGR, detected phylogenetic characteristics of species with identical mitogenome arrangements, and estimated possible time for rearrangement patterns. Our findings contribute to understanding characteristics and evolution of mitogenome rearrangement in amphibians.

## Results

### *trnM* tandem duplication of amphibian mitogenomes

Lengths of the four newly sequenced mitogenomes are 18,520 bp (*Quasipaa robertingeri*), 16,640 bp (*Limnonectes fragilis*), 18,154 bp (*Limnonectes fujianensis* (Taiwan)) and 18,293 bp (*Limnonectes fujianensis* (Fujian)), respectively, and the full range of their GC contents was relatively narrow (39.7–42.9%). The four new mitogenomes all contain a tandem duplication of *trnM* (IQMM *trn* cluster) that also occurs in other sequenced dicroglossids [14].

Among the 35 amphibian mitogenomes presenting evidence of duplication or loss of genes as well as CRs (excluding gene rearrangement with the same total number of genes), 19 species (including our four new mitogenomes) possess a tandem duplication of *trnM* (IQMM *trn* cluster) (see Table 1). These 19 species represent 16 species of Dicroglossidae, two species of Megophryidae (the non-neobatrachian frog family), and one species of the neobatrachian Ceratobatrachidae. Thus, tandem duplication of *trnM* is not exclusive to Dicroglossidae, and more than one rearrangement event is required to explain this pattern given that these families are distantly related to each other [38, 39]. The frogs *Leptolalax oshanensis* (I-Q-M-V-P-M-*nad2*) and *Mantella madagascariensis* (*nad5*-I-M-L2-P-F-*rrnS*-V1-*rrnL*-L1-T-*nad1*-M-CR) have two separate copies of *trnM* rather than a tandem duplication (Additional file 1: Table S1). The difference in position suggests that they may possess different mechanisms of occurrence [29, 31]. The amphibian species with gene duplication or loss (involved with other tRNA genes, rRNA genes, PCGs and CRs) are also shown in Additional file 1: Table S1.

**Table 1:** List of amphibian mitogenomes with two *trnM* genes in this study

Species	Taxon	Accession Nos
*Platymantis vitianus*	Anura; Ceratobatrachidae	NC_027671
*Euphlyctis hexadactylus*	Anura; Dicroglossidae	NC_014584
*Fejervarya cancrivora*	Anura; Dicroglossidae	NC_012647
*Fejervarya limnocharis*	Anura; Dicroglossidae	NC_005055
*Hoplobatrachus rugulosus*	Anura; Dicroglossidae	NC_019615
*Hoplobatrachus tigerinus*	Anura; Dicroglossidae	NC_014581
*Nanorana parkeri*	Anura; Dicroglossidae	NC_026789
*Nanorana pleskei*	Anura; Dicroglossidae	NC_016119
*Nanorana taihangnica*	Anura; Dicroglossidae	NC_024272
*Occidozyga martensii*	Anura; Dicroglossidae	NC_014685
*Quasipaa boulengeri*	Anura; Dicroglossidae	NC_021937
*Quasipaa robertingeri*	Anura; Dicroglossidae	this study
*Quasipaa spinosa*	Anura; Dicroglossidae	NC_013270
*Quasipaa yei*	Anura; Dicroglossidae	NC_024843
*Limnonectes bannaensis*	Anura; Dicroglossidae	AY899242
*Limnonectes fragilis*	Anura; Dicroglossidae	this study
*Limnonectes fujianensis* (Fujian & Taiwan)	Anura; Dicroglossidae	this study
Mantella madagascariensis*	Anura; Mantellidae	NC_007888
*Leptobrachium boringii*	Anura; Megophryidae	NC_024427
Leptolalax oshanensis*	Anura; Megophryidae	NC_020610
*Oreolalax major*	Anura; Megophryidae	NC_030605

^*^All species with two *trnM* are tandem duplication except *Leptolalax oshanensis* and* Mantella madagascariensis*

### Different patterns of mitogenome rearrangement in amphibians

**Fig. 1:**
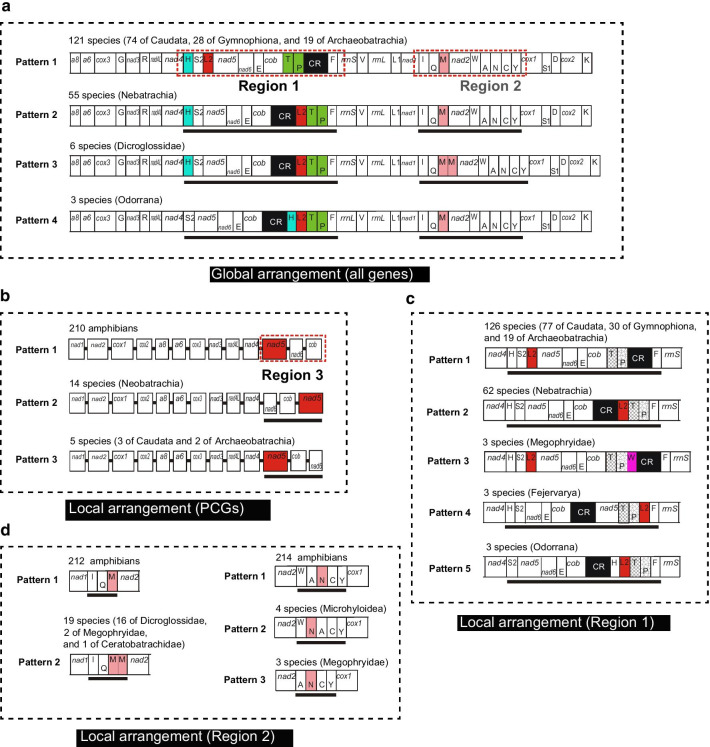
The patterns of global and local mitogenome arrangement in amphibians (occurrence frequency ≥ 3 times among sampled taxa). The number of occurrences and categorization are shown above each illustration of patterns, main rearranged genes or regions are marked with color, red dashed box or underline). **a** Global arrangement patterns involved with all mt genes. **b** Local arrangement patterns only involved with PCGs. **c** Local arrangement patterns in Region 1. **d** Local arrangement patterns of IQM region and WANCY region in Region 2. Abbreviations of mt genes follows ref. [14]

We subsequently investigated more rearrangement types of 232 amphibian complete mitogenomes filtered by available annotation information. Among them, 121 species’ mitogenomes represented typical vertebrate arrangement, and the remaining 111 species possessed 43 types of gene arrangement, when we examined the arrangements of all mitochondrial (mt) genes involved, which were defined as global arrangement. Their arrangements were summarized in Additional file 1: Figure S1. However, ignoring the species with rare arrangements (only one or two occurrences), they all belonged to major arrangements that occurred at least three times (irrespective of phylogeny) in amphibian mitogenomes, as shown in Fig. 1a. In the figure, there were only four patterns in these global arrangements. We labeled them as Patterns 1 ~ 4 in descending order of the frequency of rearrangement types. And we identified two segments that were subject to highly frequent rearrangements as Region 1 and Region 2, respectively. Between them, Region 1 covered the *nad5-nad6-cob* gene cluster and CR, and Region 2 was mainly composed of IQM *trn* cluster and WANCY *trn* cluster. Compared with typical vertebrate arrangement (Pattern 1), 55 neobatrachians shared the second most frequent pattern of mitogenome arrangement, known as typical neobatrachian arrangement, which had an LTPF *trn* cluster in their Region 1, and we found that all neobatrachians belonged to Pattern 2. Due to the existence of novel IQMM *trn* cluster in Region 2, the six species of Dicroglossidae including the new mitogenomes determined were distinguished from typical neobatrachian arrangement and formed a new pattern, Pattern 3. Pattern 4 occurred in three species of the genus *Odorrana*, whose HLTPF *trn* cluster replaced LTPF *trn* cluster of neobatrachians in Region 1.

PCGs play an important role in mitogenome rearrangement in vertebrates [40, 41]. To reduce the complexity of mitogenome rearrangement caused by RNA genes and CRs, we further investigated the local arrangement (not all genes involved) limiting analyses on only PCGs. Figure 1b shows the 3 patterns (Pattern 1 ~ 3) of PCGs arrangements (frequency ≥ 3), which only the frog *Hoplobatrachus rugulosus*, a species with double mitochondrial *nad5* genes (only present once among sampled amphibians) was excluded. Among them, 91.3% of the sampled amphibians shared the first pattern (Pattern 1) of PCGs arrangement, while the other 19 species from Anura and Caudata belonged to the remaining two patterns, implying that the probability of rearrangement involved proteins is much smaller than other genes. The major differences among the 3 patterns related to the gene orders of *nad5*, *nad6,* and *cob*. Therefore, we considered that the *nad5-nad6-cob* segment may be “an active region” of mt PCGs rearrangement, and the segment was defined as Region 3.

Figure 1c and d show the patterns of mitogenomic local arrangements that occurred at least three times in Region 1 and Region 2, respectively. The Region 1 of Fig. 1c included two extra *trn* clusters: a TPW *trn* cluster at the 5′ end of CR and a TPLF *trn* cluster at the 3′ end of CR, which was derived from 3 species of Megophryidae and 3 species of genus *Fejervarya*, respectively, compared with Fig. 1a of mitogenomic global arrangements. The left part of Fig. 1d shows that a novel local arrangement, the IQMM *trn* cluster, was derived from 19 species out of 3 families (all within Anura), namely Dicroglossidae, Megophryidae, and Ceratobatrachidae (see also results of *trnM* tandem duplication above). Similarly, we also found other rearrangement patterns in the WANCY region of Region 2, the pattern of ‘WNACY’ was present in 4 species of Microhyloidae and the pattern of ‘ANCY’ was present in 3 species of Megophryidae (whose mitogenome possessed the TPW *trn* cluster).

**Fig. 2:**
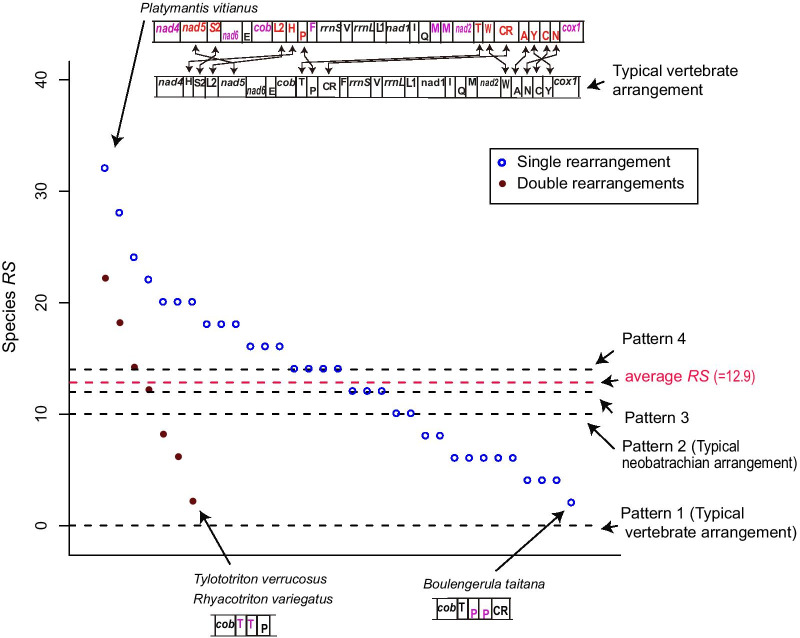
Scatter plot of *RS* of species with rare arrangements (Single rearrangement and Double rearrangements). The red dotted line represents the average *RS* of 47 species with rare arrangements; The black dotted lines indicate *RS* of species belonging to four major patterns (global arrangement). The figure also shows the species with the highest and the lowest *RS* and their rearrangement information, and the names of genes with two changed flanking genes are marked in red, the names of genes with changes of one-sided gene are marked in purple

Compared to major arrangements, 47 species with rare arrangements accounted for 42.3% of all rearranged amphibian mitogenomes including 7 pairs of species with double rearrangements (the same rearrangement type appeared only twice in all sampled species) and 33 species with single rearrangement (this rearrangement type is unique in all sampled species) (Fig. 2).

### Accurate quantification of rearrangement frequency

**Fig. 3:**
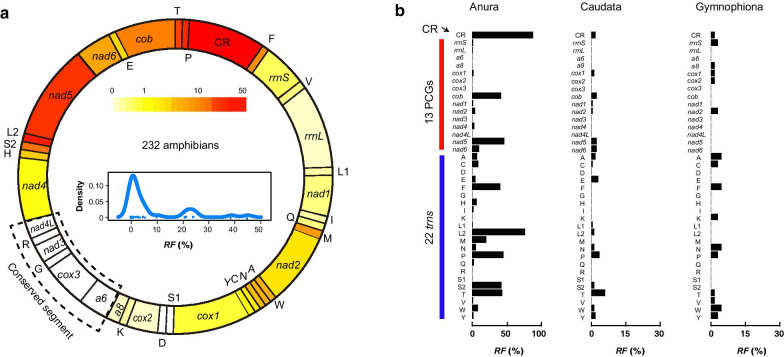
*RF* distributions of mt genes of all sampled amphibians (**a**) and species of three orders (**b**) using the qMGR method. In (**a**), the outer ring shows the mitogenome arrangement of most amphibians (namely, typical vertebrate arrangement). Linear density distribution is in its inner bottom part, and the depth of colors corresponding to the different values of *RF* are placed in its inner upper part, and the higher the value, the darker the color. The control region (CR) and *trnL2* gene are the two most frequently rearranged components. We also mark three or more contiguous conserved genes with *RF* of zero by dotted lines; In (**b**), the histograms indicates that *RF* of each gene in three separate groups: frogs, caecilians, and salamanders

qMGR [35] is a method for large-scale and rapid quantification of mitochondrial genome rearrangements. Compared with the arrangement of the reference genome, it can calculate the rearrangement score (*RS*) of each gene in each genome one by one based on the changes of genes on its both sides, and accumulate *RS* of all genes in this genome to obtain the genome’s *RS*. In a given taxonomic group, dividing the actual *RS* of a gene by its maximum possible *RS* can be used to extrapolate the relative rearrangement frequency (*RF*) of the individual gene. qMGR was used to accurately calculate the *RF* of amphibian mitochondrial genes. Figure 3a and b show the *RF* distributions of all individual genes of amphibians and their three consistent orders, respectively. As shown in Fig. 3a, *RF* of most genes were less than 10 (%), the genes with high *RF* (%, coloring deeper) were mainly concentrated in the *nad5*-CR segment and IQM-WANCY segment (namely, Region 1 and Region 2 mentioned above), consistent with previous findings for rearrangement hotspots in vertebrate mitogenomes [16, 42]. For each single gene, CR was assigned the highest *RF*(%) with a score of 45.04 by using qMGR [35], indicating that about 45% of the flanking genes of CR have been changed, that is, the changes have been accumulated more than 200 times (*trnP* and *trnF* are located at the 5′ and 3′ end of CR, respectively). *trnL2* was ranked second with a score of 38.79, and *nad5* had the highest *RF(%)* among all PCGs, with a score of 24.14. In addition, according to the definition of *RF*, if consecutive adjacent genes all have the smallest *RF* value (here 0), we believed that they themselves formed a rearranged conserved segment in the mitochondrial genome. We identified the two most conserved segments in amphibian mitogenomes as *atp6*-*cox3*-G-*nad3*-R-*nad4L* and S-D between *cox1* and *cox2.*

In Fig. 3b, there were fewer genes with a non-zero *RF* (< 50%) and with relatively low scores (< 15) in Caudata and Gymnophiona. In comparison, *RF* of most genes (27 of the total 38) in Anura were greater than zero, and some of them were greater than 30. The primary reason was that most species with rearranged mitogenomes belonged to typical neobatrachian arrangement in this group (56.3%). Also, *RF* of anuran *trnL2* and CR were both greater than 50, mainly due to the fact that their rearrangements were accompanied by > 50% changes in adjacent genes on both sides compared to typical vertebrate arrangement.

**Fig. 4:**
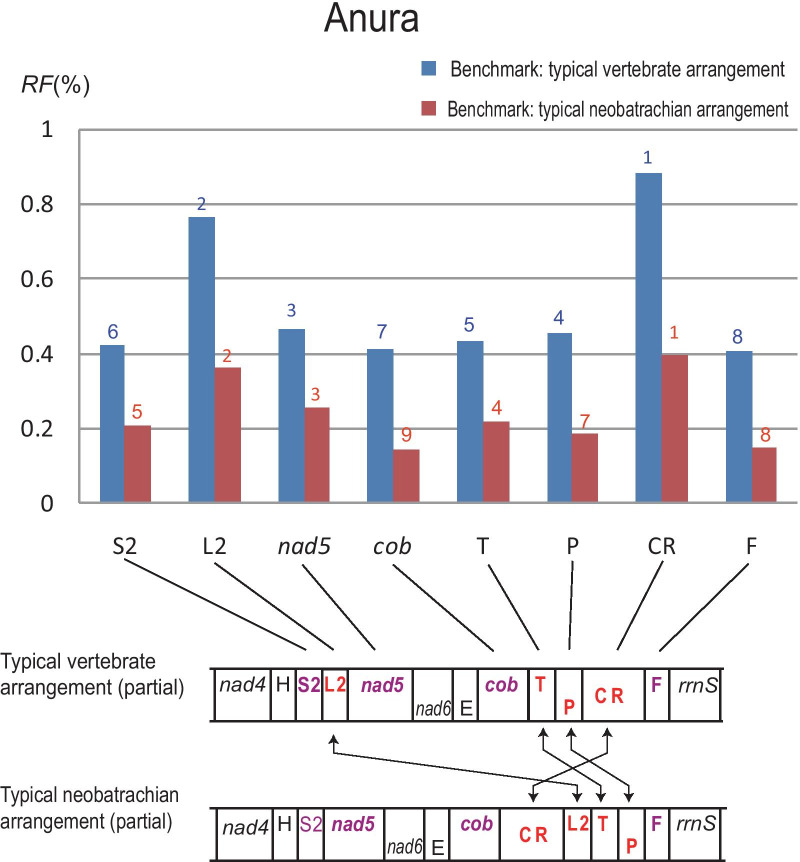
Histogram of rearranged genes’ *RF* in anuran mitogenomes based on two different benchmarks, and comparison of two typical arrangements (partial) below it. In the figure above, the number above strips represents rank of *RF* of single gene in all genes sorted in descending order. In the segments rearranged of the figure below, and the color of the gene names represents the same meaning as in Fig. 2

Considering that typical neobatrachian arrangement is the dominant gene order in frogs’ mitogenomes, we also tried to choose it as the benchmark for calculating *RF*. The results showed that the genes with changed *RF* were those rearranged genes relative to typical vertebrates arrangement (Fig. 4). Interestingly, all of their scores had declined, but the relative rank of their *RF* changes little. The top 3 of them were both CR, *trnL2*, and *nad5* in order, implying that they are the most active elements of mitogenome rearrangement in frogs.

To further investigate the rearrangement degree of species with rare arrangement, based on qMGR [35], we also calculated their species *RS,* which was the cumulative value of the rearrangement scores of all genes in the genome of a given species. As shown in Fig. 2, we found that the species with the highest *RS* is the neobtrachian frog *Platymantis vitianus* (species *RS* = 32), which had 17 genes involved in rearrangement compared with typical vertebrate arrangement. The species with the lowest scores, consisting of the salamander *Tylototriton verrucosus,* the salamander *Rhyacotriton variegatus* and the caecilian *Boulengerula taitana* (species *RS* = 2), had only one rearranged gene, which was a tandem duplcation of *trnT, trnT,* and *trnP*, respectively (Fig. 2). The average *RS* of all species with rare arrangements was 12.9, which was slightly higher than the species with typical neobatrachian arrangement (Patten 2). In addition, Fig. 2 also shows *RS* of the species belonging to the other three global arrangement patterns.

### Phylogenetic characteristics of mitogenome rearrangement in amphibians

**Fig. 5:**
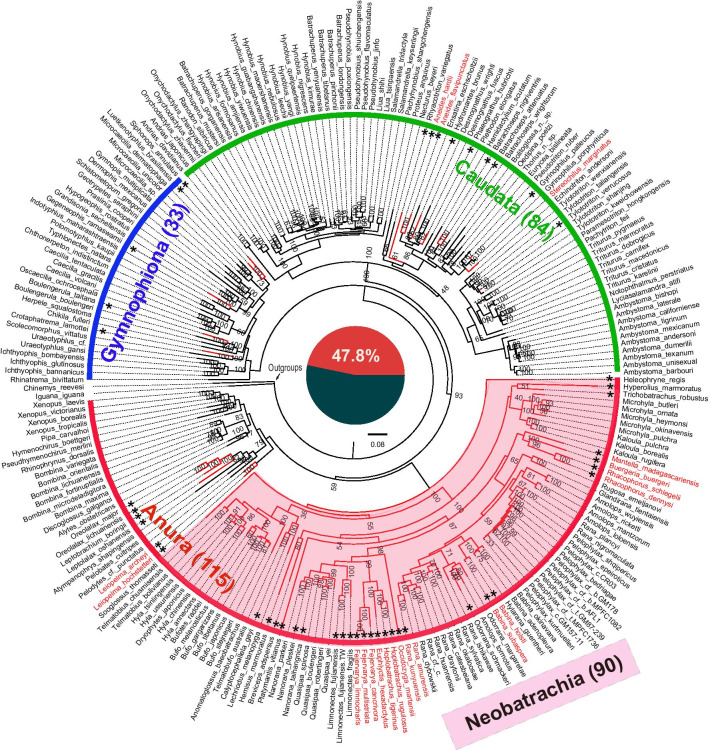
Phylogenetic tree of all amphibians studied inferred using ML method based on the nucleotide dataset of 13 mt PCGs. The tree divides extant amphibians into three major taxa (orders): Gymnophiona, Caudata and Anura (including Neobatrachia) and the number of sampled species are shown in brackets. We have marked the branches of the species with rearrangements and species names only involved with PCGs rearrangements in dark red, names of species with rare arrangement are marked with an asterisk, and a pale red background is given to Neobatrachia, the group with the most intensive rearrangements. The middle pie chart shows the proportion of sampled amphibian species with mt gene rearrangements

For phylogenetic characteristics of rearranged amphibian mitogenomes, the optimal ML tree and BI tree of 232 amphibians were shown in Fig. 5 and Additional file 1: Figure. S2, respectively, and they had similar basic topologies. In them, the monophylies of the three amphibian groups (frogs, caecilians, and salamanders) were strongly supported, as found in most phylogenetic analyses [38, 43–46]. Within frogs, the relationship of two higher-level groups, Neobatrachia and Archaeobatrachia (non-neobatrachian frogs) was consistent with most previous reports (e.g., [38, 44, 47, 48]). Overall support was high within all species. 84% of the nodes had a bootstrap value (BS) ≥ 75% (Fig. 5), and 98% had Bayesian posterior probabilities (BPPs) ≥ 0.90 (Additional file 1: Figure. S2). Our results suggested that the superfamily and family-level relationships of Neobatrachia were mostly consistent with most previous studies (i.e., superfamily Ranoidea includes Ranidae, Dicroglossidae, Microhylidae, Mantellinae, Rhacophoridae, etc.) [38, 44, 49, 50]. Additionally, BS and BPPs also strongly supported most families of salamanders and caecilians, for instance, Salamandridae, Plethodontidae, and Hynobiidae within Caudata [51], and Caeciliidae, Typhlonectidae, Ichthyophiidae within Gymnophiona [52].

Compared with other vertebrate taxa, mitogenome rearrangement of amphibians is seemingly more frequent [27], particularly in neobatrachian frogs [14, 28]. Among the 232 amphibian mitogenomes examined, there were 111 species (47.8%) with non-typical organization (red branches marked in Fig. 5). In the 111 species with rearranged mitogenomes, only 15 species were members of the 117 total species sampled of Caudata (11.9%) and Gymnophiona (15.2%), while most of them were concentrated in Anura (83.5%), especially Neobatrachia (90 species: 100%). Figure 5 also shows 20 species with rearrangements of PCGs (only the gene order of 13 PCGs in the mitogenome was considered), accounting for only 8.6% of total species (red species name marked in Fig. 5). Species with rearranged mitogenomes tended to be phylogenetically clustered whether studied on all genes (p < 1.0e–10, hypergeometric test) or PCGs (p < 0.001, hypergeometric test), suggesting that mitogenomic rearrangement of amphibians also possesses phylogenetic characteristics, similar with insects [22] and birds [53].

In Fig. 5, these species with rare arrangement were marked with asterisks. We found that they included 90% of the species with rearrangements of PCGs, which might be one of the reasons why their rearrangements were “rare”. Among the 232 species examined, 28 species belong to 22 families, each of which includes only one or two species. We also further examined whether the rarity could be due to the density of taxon sampling. The result showed that it was highly dependent on density of taxon sampling (p < 1.0e-04, hypergeometric test).

### Rearrangement time estimation of amphibian mitogenomes

Because mitogenome rearrangement of amphibians possessed phylogenetic characteristics, and the same gene arrangement among different species was generally unlikely to be the result of convergent evolution [11], we could estimate the (latest) possible occurrence time of their pattern according to the divergence time of common ancestor lineages with the same rearrangement pattern. So we referred to the literature on divergence time of different taxa of amphibians [14, 39, 43, 45], and estimated the possible occurrence time of various patterns from three rearrangement enrichment regions, namely, Region 1, Region 2 and Region 3, as shown in Fig. 1.

Compared with the older Amphioxus (*Branchiostoma floridae*) mitogenome arrangement [54], the pattern of typical amphibian arrangement was consistent with that of vertebrates, including H-S2-L2-*nad5*-*nad6*-E-*cob*-T-P-CR-F (Region 1), IQM-*nad2*-WANCY (Region 2), and *nad5*-*nad6*-*cob* (Region 3). Different from their Region 3, the pattern, *nad5-cob-nad6,* were identified in 3 caudatans and 2 archaeobatrachians. It implied that the rearrangement pattern could have occurred before the divergence of frogs and salamanders, that is, it may be earlier than 350 million years ago (Ma) [39]. Four newly sequenced species of neobatrachians, and 2 archaeobatrachians, all had the IQMM pattern (IQMM *trn* cluster), suggesting that the pattern can appear earlier than 250 Ma, before the divergence of neobatrachians and archaeobatrachians in Anura [39, 43] (Additional file 1: Figure. S3). Similarly, the pattern, H-S2-*nad5*-*nad6*-E-*cob*-CR-L2-T-P-F (from Region 1 of typical neobatrachian arrangement) may have existed in the Late Jurassic (earlier than 140 Ma) when neobatrachians appeared [14]. We also marked possible occurrence time of other rearrangement patterns in Additional file 1: Figure. S3.

According to the estimated time of the rearrangement patterns, as shown in the lower left corner of Additional file 1: Figure. S3, we speculated that the rearrangement of amphibian mitogenomes might present the following trends: (1) the *trn* genes near CR tended to be rearranged to the 3′ end of CR which was consistent with the transcription direction of protein-coding genes; (2) the *nd5* gene also had a tendency to rearrange to the 3′ end of CR.

## Discussion

### Mitogenome rearrangement and qMGR method

Just like our newly determined species, rearrangement events are often accompanied by related gene duplication as well as gene loss. Most existing models of rearrangement mechanisms often involve gene duplication and gene deletion (e.g. [17–19, 55, 56],). Among these models, TDRL (tandem duplication–random loss) model has been widely accepted to explain the rearrangement mechanism of amphibian mitochondrial genomes[34], Such as *Fejervarya limnocharis* [57], *L. bannaensis*[29], *Hoplobatrachus tigerinus* [58], *Leiopelma archeyi* [59]. With TDRL model, Xia et al. [34] perfectly explained the rearrangement events within the species. TDNL (tandem duplication and non-random loss) is also another similar model involving gene duplication and gene loss, and it has been used to rationally explain the rearrangement of the millipedes (*Narceus annularus* and *Thyropygus sp*.) [18]. In addition, DMNR (the Dimer-Mitogenome and Non-Random Loss) and DRRL (the Double Replications and Random Loss) have also been proposed to explain the course of the rearrangement in two flatfish mitogenomes (*Crossorhombus azureus* and *Samariscus latus*) [19, 60].

Our research has found that *trn* genes were more prone to rearrangement than other genes, and some *trn* genes (such as *trnL2, trnT,* and *trnP*) preferred to turn to the 3′ end of CR. Satoh et al*.* [61] proposed that there was a significant correlation between *trn* gene position (that is, *trn* gene order) and codon usage in vertebrate mitogenomes, and the closer to the 3′ end of CR the *trn* gene was, the higher its usage was, inferring that the mt gene arrangement of vertebrates is affected by translation constraints, which may help maintain the gene order for a long time. However, Xia et al*.* [28] claimed that the correlation was not significant for them of typical neobatrachian mitogenome. Furthermore, Xia et al*.* [34] also believed that the gene distributions of rearranged frogs were related to their non-adaptive forces. Therefore, the mechanism of *trn* gene order is still under debate in amphibian mitogenomes.

Nevertheless, due to the relatively rarity of rearrangements and their constitution of useful synapomorphies, they may provide more useful information for phylogenetic inference with increasing difficulty of their own research [16, 28]. A large number of studies have been carried out to clarify possible units of rearrangement and possible evolutionary processes between two rearranged mitogenomes (e.g. [17, 22, 23, 29]). qMGR has more flexibility than other rearrangement analysis algorithms (e.g., CREx [25], TreeRex [62], amGRP [63] and GRAPPA [64]), and can analyze mitogenomes (or mt gene fragments) of different numbers and different rearrangement types, even other circular genomes such as chloroplasts. In the absence of prior knowledge, within a certain group, qMGR [35] was able to filter out highly rearranged genes and genomes, which would contribute to the study of the choice of rearrangement models and rearrangement pathways.

### Mitogenome rearrangement and phylogenetic analysis

Many previous rearrangement studies were often accompanied by phylogenetic analysis (e.g., [14, 32, 34, 50]), which helped to speculate on the evolution of rearrangement events. In our study, we reconstructed two phylogenetic trees (BI tree and ML tree). We found that the phylogenetic relationship of most neobtrachians was consistent with previous studies (e.g., [38, 43, 44, 49, 65]), the relationship between teresomatans and all other caecilians was in full agreement with previous reports (e.g. [44, 49, 52],), and our trees also supported a sister-group relationship between frogs and salamanders (the Batrachia hypothesis), as found in most previous studies(e.g. [44, 52, 66],). In addition, the relationship between Leiopelmatidae and all other frogs was compatible with previous reports in BI tree (e.g. [50, 59, 67]). The sister relationships of Pipoidea and Discoglossoidea were consistently supported (BPPs = 1, BS = 83%), however, some recent studies did not support the view (e.g. [38, 44, 50]). The inconsistent results may be affected by different species and selected molecular markers. But these subtle differences did not change the overall distribution of amphibian rearrangement types in the phylogenetic trees. Based on the results of the phylogenetic study above, species with the same pattern of mitogenome rearrangement mostly belonged to closely related taxa. Just like the previous reports of avian mitogenome rearrangement [53], the phylogenetic characteristics of rearrangement were further interpreted that they could originate from a common ancestor and were then retained during subsequent lineage diversification. Therefore, this makes it possible to use the divergence time of ancestral species with common mitogenome arrangement to estimate the occurrence time of rearrangement patterns.

### Non-coding regions of mitogenome rearrangement

In the mitochondrial genomes, two non-coding regions, CR and O_L_, play important roles in the rearrangement studies [16, 42]. The duplicated CR and mitochondrial gene rearrangement have been found in many parrot species [68]. Our study showed that CR was the most active element in the mitogenome rearrangement of amphibians. When we disregarded it in the study of PCGs rearrangements, the results may have missed some information. For example, *nad6-cob*-CR-*nad5* of neobtrachian frog *Buergeria buergeri* [69] and *nad6-cob*-CR-*nad5*-CR of neobtrachian frog *R. schlegelii* [32] were treated as the same arrangement type.

Unfortunately, we found that a few species in the GenBank database have incomplete or even incorrect annotations for O_L_ (as found by the references [70]). So we ignored this important element in the WANCY region, which was considered as a hotspot for rearrangement [16, 42]. This made us unable to distinguish between ACW-O_L_-NY of marsupial *Trichosurus vulpecula* [71] and A-O_L_-CWNY of caecilian *Siphonops paulensis* [16], and between WA-O_L_-NCY and WAN-O_L_-CY of neobtrachian frog *Q. boulengeri* (intraspecific rearrangement of mitogenome) [34]. These implied that the components examined in the study of gene rearrangements had a great influence on the results. In fact, some reports have found that O_L_ is absent in birds, crocodiles, fish, scorpions, etc. [42, 72, 73]. However, in vertebrates with mitochondrial O_L_, the WANCY region of amphibians possessed the most frequent rearrangements, which also involved gene duplication, gene loss, and pseudogenes (e.g., [16, 29, 34, 42, 50]).

## Conclusion

In this study, we first examined the characteristics of *trnM* tandem duplication in four newly sequenced Dicroglossidae mitogenomes as well as in other amphibian taxa, and found that it was not an exclusive feature of Dicroglossidae. We then applied qMGR for calculating *RS* and *RF* of each single mt gene, and screened out high-frequency genes and conservative genes involved in different taxa of amphibian mitogenome rearrangement. Based on phylogenetic analysis, we found that mitogenomic rearrangement of amphibians possessed phylogenetic implications, and were concentrated in Neobatrachia. Still, typical vertebrate arrangement was the most dominant type of arrangements for amphibians. In addition, qMGR can also obtain *RS* of species with rare arrangements, which can be used to measure the rearrangement degree of a single species. This was a systematic survey of the rearrangement of the amphibian mitochondrial genome and its evolution. Nevertheless, as the currently available data was less than 5% of the total number of known species, our results may still lack representativeness and integrity. More data and more in-depth analysis will help us figure out thoroughly the rearrangement characteristics of amphibians.

## Methods

### Determination and analysis of 4 new mitogenomes

Samples of four dicroglossid frogs *Q. robertingeri* (code Zhang-YBJW031), *L. fragilis* (code DT-CP005), *L. fujianensis* (Taiwan population) (code Zhang-TWDT034) and *L. fujianensis* (Fujian population) (code DT-FJ002) were collected from Sichuan, Hainan, Taiwan and Fujian province in China, respectively. Total DNA was extracted from their fresh muscle tissues after the frogs were euthanized using 0.5% MS-222. Then tissues of the first two were stored at − 80 °C at the College of Life Sciences, Anhui Normal University, China, and the latter two were stored at − 20 °C at the School of Bioengineering, Huainan Normal University, China. In accordance with Regulation for the collection of genetic resources of China (HJ 628-2011), we collected all laboratory animals, and animal experiments and follow-up disposal were carried out based on Regulations for the management of laboratory animals in Huainan Normal University (2015). We designed multiple primer pairs (Additional file 1: word S1) based on sequence alignments of mitochondrial genes from closely related species [29, 57, 74], and carried out shotgun sequencing and assembling. We identified genomic components by the MITOS2 (http://mitos.bioinf.uni-leipzig.de/index.py) [75], tRNAscan-SE (http://lowelab.ucsc.edu/tRNAscan-SE/) [76], and manual sequence comparison [2, 37]. Base composition was determined using DNASTAR (www.dnastar.com).

### Preparation of rearrangement data

We downloaded sequences data of amphibian mitogenomes from NCBI: Organelle Genome Resource (http://www.ncbi.nlm.nih.gov/genome/organelle/) on May 10, 2019. In total, we retrieved mitogenomes of 228 species (in addition to the four species newly sequenced here) by filtering out the data without complete annotation information. We then extracted location information of genes and their nucleotide sequences for subsequent rearrangement analysis and phylogeny using designed Perl and R scripts.

### Different rearrangement patterns

To investigate major patterns of amphibian mitogenome rearrangement, we sorted all genes based on their position information in the genome. When starting with the same gene, we merged and counted species with identical gene orders. We defined the arrangement involved with all mt genes (37 genes + CR in this study) as a global arrangement, and those of not all genes were called local arrangements. We surveyed major arrangement whose the cumulative number with the same arrangements was ≥ 3 times, For the type of arrangement less than or equal to two times, we called it "rare arrangement". We further subdivided the high-frequency rearrangement regions in the global arrangements or local arrangements into different regions, which also belonged to local arrangements. To facilitate research, we also numbered the different arrangement types of these major arrangements into multiple patterns.

### Precise quantification of rearranged genes and genomes

qMGR can be used to accurately calculate the rearrangement frequency of each single gene or each single genome within a given taxonomic group [35]. When a reference arrangement (benchmark) of mitogenome was selected, it can accumulate changes in genes at the two nearest flanking positions of a gene to be tested and give the gene a score. Based on this principle, qMGR can calculate the rearrangement score (*RS*) of a complete mitogenome and the relative rearrangement frequency (*RF*) of individual genes within a certain group (e.g., neobatrachians, anurans or amphibians) (referring to reference [35] and its website for more details on the method). We chose typical vertebrate arrangement as a benchmark for the comprehensive analysis of mitochondrial genome rearrangement in amphibians, and also chose typical neobatrachian arrangement for comparative analysis of *RF.* In the calculation process, we regarded the CR as a single gene, while ignored pseudogenes and the origin of L-strand replication (O_L_) for their incomplete annotations.

### Phylogenetic analysis and rearrangement time estimation

We performed phylogenetic analysis using maximum likelihood (ML) [77] and Bayesian inference (BI) [78] methods based on the combined nucleotide dataset of 13 PCGs of 232 species (including 43 families, about half of them contain only 1–2 species) as well as two outgroup species (turtle: *Chinemys reevesi*, NC_006082, and iguana: *Iguana iguana* NC_002793). Multiple sequence alignment was carried out using MAFFT 7.2 [79]. The substitution saturation analyses for each codon positions of each gene were assessed by using DAMBE 7.2.25 [80]. And we detected the saturation on their third codon positions. The information of substitution saturation for all codon positions of 13 genes could be seen in Additional file 1: Table S2. Therefore, phylogenetic analysis was only based on their first two codon positions. For BI analysis, the best schemes for partition and substitution models (Additional file 1: Table S3) were determined in PartitionFinder version 2.1.1 [81] according to the Bayesian Information Criterion (BIC) and greedy search algorithm. Then the BI analysis was implemented in MrBayes v.3.2.7a [82, 83]. We ran four Markov chains for 5 × 10^7^ generations (sampling every 1000 generations) and calculated a 50% majority-rule consensus tree and Bayesian posterior probabilities (BPPs) after discarding the initial 25% trees as burn-in. All MCMC runs were repeated twice to avoid spurious results. Furthermore, the convergence of the MrBayes analyses was checked with Tracer 1.7.1 [84]. Subsequently, the ML tree was inferred with RAxML v.8.2.12 [85] using the GTRGAMMA model, 100 starting trees and 1000 bootstrap replicates to assess node support [86]. After that, the ML bootstrap convergence test was carried out with parameter "-I autoMRE".

To estimate the possible occurrence time of gene rearrangement, we referred directly to the results of the studies on divergence time estimations [14, 39, 43, 45]. Based on the divergence timetable of amphibians and related taxa, we estimated the latest possible occurrence time of the different rearrangement patterns.

## Supplementary Information

**Additional file 1: Table S1.** Amphibian species with mt gene duplication or loss in this study. **Figure S1.** Mitogenomic rearrangement patterns of all amphibian species investigated. The numbers in first column indicated the occurrence frequencies of patterns (a number less than three is defined as a rare arrangement in this study). **Figure S2.** Phylogenetic tree of all amphibians studied using BI method based on the nucleotide dataset of 13 mt PCGs. The number of species sampled for each lineage is shown in brackets. We marked the branches of the species with mt gene rearrangements and the names of species only involved with PCGs rearrangements in dark red, and a pale red background was set on the branch of neobatrachians, the group with the most intensive rearrangements. **Figure S3.** Possible occurrence time and trends of local rearrangement patterns of mitogenomes according to divergence times of their ancestors. The possible trends were shown in the box at lower left. **Word S1.** Amplification scheme and primers sequences for sequencing four mitochondrial genomes in this study. **Table S2.** Determination of substitution saturation for each codon position of each gene. **Table S3.** The best partitioning scheme selected by PartitionFinder for different data matrices.

## Data Availability

The source code of qMGR was freely available under GNU GPL at https://github.com/zhanglab2019/qMGR, and its web service was available at http://qmgr.hnnu.edu.cn/. The four complete sequences of our newly identified frogs were submitted to GenBank (https://www.ncbi.nlm.nih.gov/) with accession numbers KY441640 (*Q. robertingeri*), AY899241 (*L. fragilis*), MF678821 (*L. fujianensis* (Taiwan)) and AY974191 (*L. fujianensis* (Fujian)), respectively. We downloaded sequences data of amphibian mitogenomes from NCBI: Organelle Genome Resource (http://www.ncbi.nlm.nih.gov/genome/organelle/).

## Abbreviations

*a6*, ATP6: ATP synthase subunit 6
*a8,* ATP8: ATP synthase subunit 8
BI: Bayesian inference
BIC: Bayesian Information Criterion
BPPs: Bayesian posterior probabilities
cox *1–3*, COX1-3: Cytochrome c oxidase subunit I–III
CR, D-loop: Control regions
*cob,* Cytb: Cytochrome *b*
IQM(M): *trnI, trnQ, trnM* (*trnM*)
LTPF: *trnL* (CUN), *trnT*, *trnP*, and *trnF*
KS test: Kolmogorov–Smirnov test
Ma: Million years ago
ML: Maximum likelihood
*mt*: Mitochondrial
*nad1-6, 4L,* ND1-6, 4L: NADH dehydrogenase subunit 1–6, 4 L
NJ: Neighbor-joining method
O_L_: The origin of light strand replication
PCR: Polymerase chain reaction
qMGR: Quantifying mitogenome rearrangements
*RF*: Rearrangement frequency
*RS*: Rearrangement score
TDRL: Tandem duplication and random loss
WANCY: *trnW, trnA, trnN,* OL, *trnC,* and *trnY*
tRNA, *trn*: Transfer ribonucleic acid
*trnL1*: *trnL* (UUR)
*tCL2*: *trnL* (CUN)
*trnS1*: *trnS* (UCN)
*trnS2*: *trnS* (AGY)
