# Supplementary material for: Quantification and evolution of mitochondrial genome rearrangement in Amphibians
Source: BMC Ecol Evol. 2021 Feb 9;21:19. doi: 10.1186/s12862-021-01755-3 (PMC7871395; doi:10.1186/s12862-021-01755-3)
Supplement: Supplementary file 1 — Additional file 1: Table S1. Amphibian species with mt gene duplication or loss in this study. Figure S1. Mitogenomic rearrangement patterns of all amphibian species investigated. The numbers in first column indicated the occurrence frequencies of patterns (a number less than three is defined as a rare arrangement in this study). Figure S2. Phylogenetic tree of all amphibians studied using BI method based on the nucleotide dataset of 13 mt PCGs. The number of species sampled for each lineage is shown in brackets. We marked the branches of the species with mt gene rearrangements and the names of species only involved with PCGs rearrangements in dark red, and a pale red background was set on the branch of neobatrachians, the group with the most intensive rearrangements. Figure S3. Possible occurrence time and trends of local rearrangement patterns of mitogenomes according to divergence times of their ancestors. The possible trends were shown in the box at lower left. Word S1. Amplification scheme and primers sequences for sequencing four mitochondrial genomes in this study. Table S2. Determination of substitution saturation for each codon position of each gene. Table S3. The best partitioning scheme selected by PartitionFinder for different data matrices. [file 12862_2021_1755_MOESM1_ESM.pdf]

Additional file 1: Table S1. Amphibian species with mt gene duplication or loss in this study.

Type	Loss(-) or duplication(+)	Species	Taxon	Accession
tRNA gene	F(-1)	*Gegeneophis ramaswamii*	Indotyphlidae	NC_006301
	H(-1)	*Odorrana schmackeri*	Anura; Ranidae.	NC_027827
	T(-1), M(+1)	*Nanorana taihangnica*	Anura; Dicroglossidae.	NC_024272
	M(+1)	*Leptolalax oshanensis**	Anura; Megophryidae.	NC_020610
	K(-1), F(+1), P(+1), T(+1)	*Crotaphatrema lamottei*	Gymnophiona; Scolecomorphidae.	NC_019596
	E(+1)	*Amolops ricketti*	Anura; Ranidae.	NC_023949
	L1(+1), P(+1)	*Aneides hardii*	Caudata; Plethodontidae.	NC_006338
	F(+2)	*Breviceps adspersus*	Anura; Brevicipitidae.	NC_023379
	M(+1)	*Platymantis vitianus*	Anura; Ceratobatrachidae.	NC_027671
		Euphlyctis hexadactylus	Anura; Dicroglossidae.	NC_014584
		Fejervarya cancrivora	Anura; Dicroglossidae.	NC_012647
		Fejervarya limnocharis	Anura; Dicroglossidae.	NC_005055
		Hoplobatrachus rugulosus	Anura; Dicroglossidae.	NC_019615
		Hoplobatrachus tigerinus	Anura; Dicroglossidae.	NC_014581
		Nanorana parkeri	Anura; Dicroglossidae.	NC_026789
		Nanorana pleskei	Anura; Dicroglossidae.	NC_016119
		Occidozyga martensii	Anura; Dicroglossidae.	NC_014685
		Quasipaa boulengeri	Anura; Dicroglossidae.	NC_021937
		Quasipaa robertingeri	Anura; Dicroglossidae.	this study
		Quasipaa spinosa	Anura; Dicroglossidae.	NC_013270
		Quasipaa yei	Anura; Dicroglossidae.	NC_024843
		Mantella madagascariensis *	Anura; Mantellidae.	NC_007888
		Leptobrachium boringii	Anura; Megophryidae.	NC_024427
		Limnonectes bannaensis §	Anura; Dicroglossidae.	AY899242
		Limnonectes fragilis§	Anura; Dicroglossidae.	this study
		Limnonectes fujianensis (Fujian/Taiwan)§	Anura; Dicroglossidae.	this study
		Oreolalax major	Anura; Megophryidae.	NC_030605
	P(+1)	*Boulengerula taitana*	Gymnophiona; Herpelidae.	NC_020154
	S1(+1)	*Batrachoseps nigriventris*	Caudata; Plethodontidae.	NC_028184
	T(+1)	*Rhyacotriton variegatus*	Caudata; Rhyacotritonidae.	NC_006331
		Tylototriton verrucosus	Caudata; Salamandridae.	NC_017871
protein-coding gene	*nad5* (+1)	*Hoplobatrachus rugulosus*	Anura; Dicroglossidae.	NC_019615
rRNA gene	12S(+1)	*Breviceps adspersus*	Anura; Brevicipitidae.	NC_023379
CR	CR(+1)	*Trichobatrachus robustus*	Anura; Arthroleptidae.	NC_023382
		Euphlyctis hexadactylus	Anura; Dicroglossidae.	NC_014584
		Hoplobatrachus rugulosus	Anura; Dicroglossidae.	NC_019615
		Hoplobatrachus tigerinus	Anura; Dicroglossidae.	NC_014581
		Hyperolius marmoratus	Anura; Hyperoliidae.	NC_023381
		Leiopelma hochstetteri	Anura; Leiopelmatidae.	NC_027072
		Mantella madagascariensis	Anura; Mantellidae.	NC_007888
		Rana amurensis	Anura; Ranidae.	NC_030042
		Rana kunyuensis	Anura; Ranidae.	NC_024548

	Rhacophorus schlegelii	Anura; Rhacophoridae.	NC_007178
	Gyrinophilus palleucus	Caudata; Plethodontidae.	NC_028297
	Aneides hardii	Caudata; Plethodontidae.	NC_006338
	Crotaphatrema lamottei	Gymnophiona; Scolecomorphidae.	NC_019596

*All species with M(+1) are tandem duplication except *Leptolalax oshanensis* and *Mantella madagascariensis;*

§ Due to the complexity, other rearrangements of *Limnonectes* are not shown.

121 CR-F-12S-V-16S-L-ND1-I-Q-M-ND2-W-A-N-C-Y-COX1-S-D-COX2-K-ATP8-ATP6-COX3-G-ND3-R-ND4L-ND4-H-S-L-ND5-ND6-E-CYTB-T-P
54 CR-L-T-P-F-12S-V-16S-L-ND1-I-Q-M-ND2-W-A-N-C-Y-COX1-S-D-COX2-K-ATP8-ATP6-COX3-G-ND3-R-ND4L-ND4-H-S-ND5-ND6-E-CYTB
6 CR-L-T-P-F-12S-V-16S-L-ND1-I-Q-M-M-ND2-W-A-N-C-Y-COX1-S-D-COX2-K-ATP8-ATP6-COX3-G-ND3-R-ND4L-ND4-H-S-ND5-ND6-E-CYTB
3 CR-H-L-T-P-F-12S-V-16S-L-ND1-I-Q-M-ND2-W-A-N-C-Y-COX1-S-D-COX2-K-ATP8-ATP6-COX3-G-ND3-R-ND4L-ND4-S-ND5-ND6-E-CYTB
2 CR-F-12S-V-16S-L-ND1-I-Q-M-ND2-W-A-N-C-Y-COX1-S-D-COX2-K-ATP8-ATP6-COX3-G-ND3-R-ND4L-ND4-H-S-L-ND5-ND6-E-CYTB-T-T-P
2 CR-F-12S-V-16S-L-ND1-I-Q-M-ND2-W-A-N-C-Y-COX1-S-D-COX2-K-ATP8-ATP6-COX3-G-ND3-R-ND4L-ND4-H-S-L-ND5-CYTB-T-ND6-E-P
2 CR-S-ND5-E-L-T-P-F-12S-V-16S-L-ND1-I-Q-M-ND2-W-A-N-C-Y-COX1-S-D-COX2-K-ATP8-ATP6-COX3-G-ND3-R-ND4L-ND4-H-ND6-CYTB
2 CR-F-12S-V-16S-L-ND1-I-Q-M-M-ND2-A-N-C-Y-COX1-S-D-COX2-K-ATP8-ATP6-COX3-G-ND3-R-ND4L-ND4-H-S-L-ND5-ND6-E-CYTB-T-P-W
2 CR-ND5-T-P-L-F-12S-V-16S-L-ND1-I-Q-M-M-ND2-W-A-N-C-Y-COX1-S-D-COX2-K-ATP8-ATP6-COX3-G-ND3-R-ND4L-ND4-H-S-ND6-E-CYTB
2 CR-L-ND5-CR-T-P-F-12S-V-16S-L-ND1-I-Q-M-ND2-W-A-N-C-Y-COX1-S-D-COX2-K-ATP8-ATP6-COX3-G-ND3-R-ND4L-ND4-H-S-ND6-E-CYTB
2 CR-P-CR-ND5-L-T-F-12S-V-16S-L-ND1-I-Q-M-M-ND2-W-A-N-C-Y-COX1-S-D-COX2-K-ATP8-ATP6-COX3-G-ND3-R-ND4L-ND4-H-S-ND6-E-CYTB
1 CR-P-T-L-F-12S-V-16S-L-ND1-I-Q-M-ND2-CR-W-N-A-C-Y-COX1-S-D-COX2-K-ATP8-ATP6-COX3-G-ND3-R-ND4L-ND4-H-S-ND5-ND6-E-CYTB
1 CR-F-12S-V-16S-L-ND1-I-Q-M-ND2-A-C-W-N-Y-COX1-S-D-COX2-K-ATP8-ATP6-COX3-G-ND3-R-ND4L-ND4-H-S-L-ND5-ND6-E-CYTB-T-P
1 CR-ND5-I-M-L-P-F-12S-V-16S-L-T-ND1-M-CR-Q-ND2-W-A-N-C-Y-COX1-S-D-COX2-K-ATP8-ATP6-COX3-G-ND3-R-ND4L-ND4-H-S-ND6-E-CYTB
1 CR-F-12S-V-16S-L-ND1-I-Q-M-ND2-N-C-Y-W-A-COX1-S-D-COX2-K-ATP8-ATP6-COX3-G-ND3-R-ND4L-ND4-H-S-L-ND5-ND6-E-CYTB-T-P
1 CR-ND5-T-P-F-12S-V-16S-L-ND1-I-Q-M-M-ND2-W-A-N-C-Y-COX1-S-D-COX2-K-ATP8-ATP6-COX3-G-ND3-R-ND4L-ND4-H-S-ND6-E-CYTB-CR-ND5-L
1 CR-ND6-E-P-F-12S-V-16S-L-ND1-I-Q-M-ND2-W-A-N-C-Y-COX1-S-D-COX2-K-ATP8-ATP6-COX3-G-ND3-R-ND4L-ND4-H-S-L-ND5-CYTB-T
1 CR-P-L-T-F-12S-V-16S-L-ND1-I-Q-M-ND2-W-N-A-C-Y-COX1-S-D-COX2-K-ATP8-ATP6-COX3-G-ND3-R-ND4L-ND4-H-S-ND5-ND6-E-CYTB
1 CR-ND5-T-P-L-F-12S-V-16S-L-ND1-I-Q-M-ND2-W-A-N-C-Y-COX1-S-D-COX2-K-ATP8-ATP6-COX3-G-ND3-R-ND4L-ND4-H-S-ND6-E-CYTB
1 CR-A-Y-C-N-COX1-S-D-COX2-K-ATP8-ATP6-COX3-G-ND3-R-ND4L-ND4-ND5-S-ND6-E-CYTB-L-H-P-F-12S-V-16S-L-ND1-I-Q-M-M-ND2-T-W
1 CR-T-F-12S-L-P-F-H-S-F-12S-V-16S-L-ND1-I-Q-M-ND2-W-N-A-C-Y-COX1-S-D-COX2-K-ATP8-ATP6-COX3-G-ND3-R-ND4L-ND4-ND5-ND6-E-CYTB
1 CR-F-12S-16S-L-ND1-I-Q-M-V-P-M-ND2-A-N-C-Y-COX1-S-D-COX2-K-ATP8-ATP6-COX3-G-ND3-R-ND4L-ND4-H-S-L-ND5-ND6-E-CYTB-T
1 CR-L-F-12S-V-16S-L-ND1-I-Q-M-ND2-W-N-A-C-Y-COX1-S-D-COX2-K-ATP8-ATP6-COX3-G-ND3-R-ND4L-ND4-H-CR-T-P-S-ND5-ND6-E-CYTB
1 CR-ND5-T-L-P-F-12S-V-16S-L-ND1-I-Q-M-ND2-W-A-N-C-Y-COX1-S-D-COX2-K-ATP8-ATP6-COX3-G-ND3-R-ND4L-ND4-H-S-ND6-E-CYTB
1 CR-F-12S-V-16S-L-ND1-I-Q-M-ND2-S-W-A-N-C-Y-COX1-S-D-COX2-K-ATP8-ATP6-COX3-G-ND3-R-ND4L-ND4-H-S-L-ND5-ND6-E-CYTB-T-P
1 CR-L-ND1-I-Q-M-ND2-W-A-N-C-Y-COX1-S-D-COX2-K-ATP8-ATP6-COX3-G-ND3-R-ND4L-ND4-H-S-L-ND5-CYTB-T-E-P-CR-F-12S-V-16S-L-ND6-E-P
1 CR-F-12S-V-16S-L-ND1-I-Q-M-ND2-W-A-N-C-Y-COX1-S-D-COX2-K-ATP8-ATP6-COX3-G-ND3-R-ND4L-ND4-H-S-L-ND5-T-ND6-E-CYTB-P
1 CR-L-T-P-F-12S-V-16S-L-ND1-I-Q-M-ND2-W-A-N-C-Y-COX1-S-D-COX2-K-ATP8-ATP6-COX3-G-ND3-R-ND4L-ND4-S-ND5-ND6-E-CYTB
1 CR-12S-V-16S-L-ND1-I-Q-M-ND2-W-A-N-C-Y-COX1-S-D-COX2-K-ATP8-ATP6-COX3-G-ND3-R-ND4L-ND4-H-S-L-ND5-ND6-E-CYTB-T-P
1 CR-L-T-F-12S-V-16S-L-ND1-I-Q-M-M-ND2-W-COX1-S-D-COX2-K-ATP8-ATP6-COX3-G-ND3-R-ND4L-ND4-H-S-ND5-ND6-E-CYTB

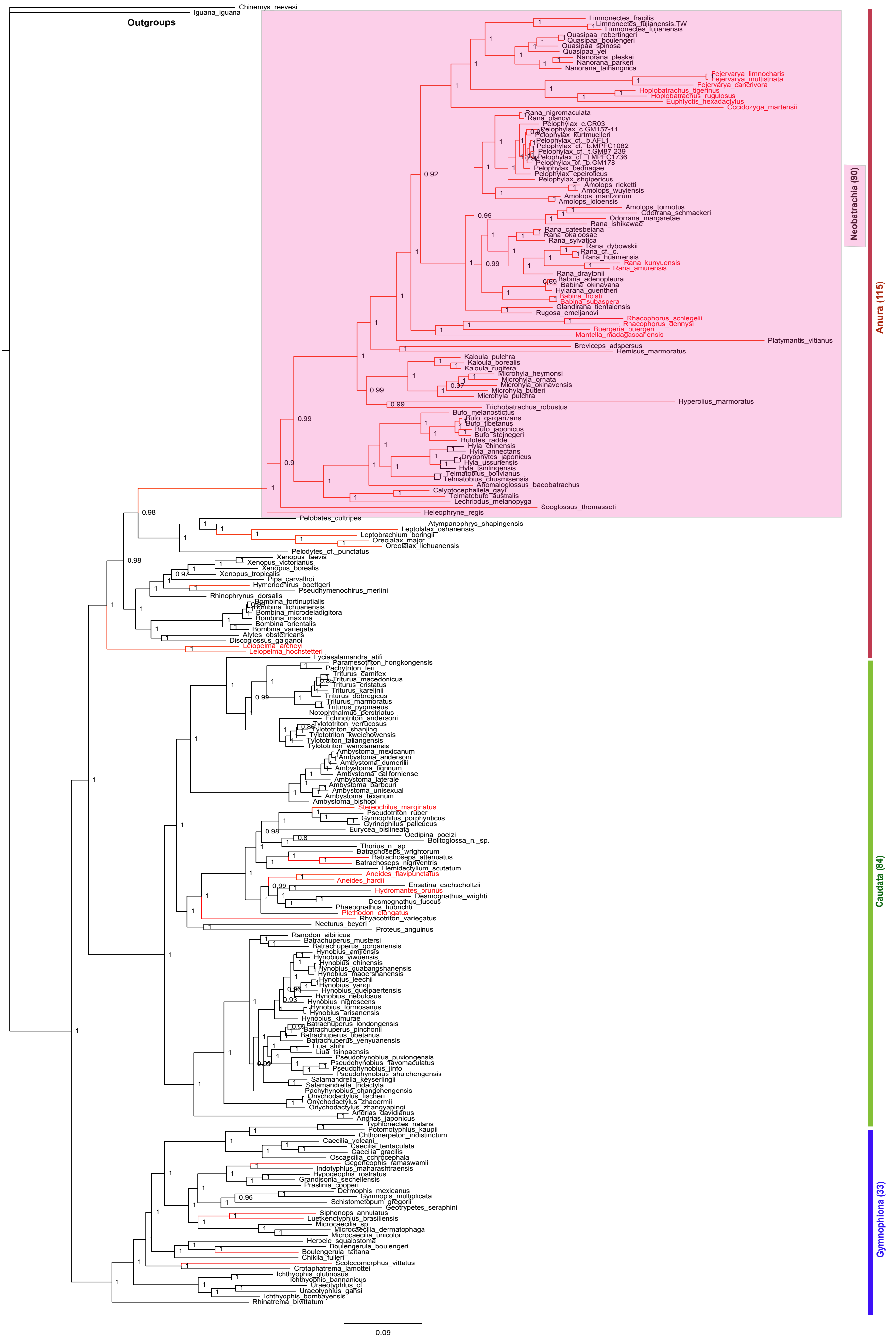
Outgroups
Neobatrachia (90)
Anura (115)
Caudata (84)
Gymnophiona (33)
0.09
Chinemys_reevesi
Iguana_iguana
Limnonectes_fragilis
Limnonectes_fujianensis.TW
Limnonectes_fujianensis
Quasipaa_robertingeri
Quasipaa_boulengeri
Quasipaa_spinosa
Quasipaa_yei
Nanorana_pleskei
Nanorana_parkeri
Nanorana_taihangnica
Fejervarya_limnocharis
Fejervarya_multistriata
Fejervarya_cancrivora
Hoplobatrachus_tigerinus
Hoplobatrachus_rugulosus
Euphlyctis_hexadactylus
Occidozyga_martensii
Rana_nigromaculata
Rana_plancyi
Pelophylax_c.CR03
Pelophylax_c.GM157-11
Pelophylax_kurtmuelleri
Pelophylax_cf._b.AFL1
Pelophylax_cf._b.MPFC1082
Pelophylax_cf._t.GM87-239
Pelophylax_cf._t.MPFC1736
Pelophylax_cf._b.GM178
Pelophylax_bedriagae
Pelophylax_epeiroticus
Pelophylax_shqipericus
Amolops_ricketti
Amolops_wuyiensis
Amolops_mantzorum
Amolops_loloensis
Amolops_tormotus
Odorrana_schmackeri
Odorrana_margaretae
Rana_ishikawae
Rana_catesbeiana
Rana_okaloosae
Rana_sylvatica
Rana_dybowskii
Rana_cf._c.
Rana_huanrensis
Rana_kunyuensis
Rana_amurensis
Rana_draytonii
Babina_adenopleura
Babina_okinavana
Hylarana_guentheri
Babina_holsti
Babina_subaspera
Glandirana_tientaiensis
Rugosa_emeljanovi
Rhacophorus_schlegelii
Rhacophorus_dennysi
Buergeria_buergeri
Mantella_madagascariensis
Platymantis_vitianus
Breviceps_adspersus
Hemisus_marmoratus
Kaloula_pulchra
Kaloula_borealis
Kaloula_rugifera
Microhyla_heymonsi
Microhyla_ornata
Microhyla_okinavensis
Microhyla_butleri
Microhyla_pulchra
Hyperolius_marmoratus
Trichobatrachus_robustus
Bufo_melanostictus
Bufo_gargarizans
Bufo_tibetanus
Bufo_japonicus
Bufo_stejnegeri
Bufotes_raddei
Hyla_chinensis
Hyla_annectans
Dryophytes_japonicus
Hyla_ussuriensis
Hyla_tsinlingensis
Telmatobius_bolivianus
Telmatobius_chusmisensis
Anomaloglossus_baeobatrachus
Calyptocephalella_gayi
Telmatobufo_australis
Lechriodus_melanopyga
Sooglossus_thomasseti
Heleophryne_regis
Pelobates_cultripes
Atympanophrys_shapingensis
Leptolalax_oshanensis
Leptobrachium_boringii
Oreolalax_major
Oreolalax_lichuanensis
Pelodytes_cf._punctatus
Xenopus_laevis
Xenopus_victorianus
Xenopus_borealis
Xenopus_tropicalis
Pipa_carvalhoi
Hymenochirus_boettgeri
Pseudhymenochirus_merlini
Rhinophrynus_dorsalis
Bombina_fortinuptialis
Bombina_lichuanensis
Bombina_microdeladigitora
Bombina_maxima
Bombina_orientalis
Bombina_variegata
Alytes_obstetricans
Discoglossus_galganoi
Leiopelma_archeyi
Leiopelma_hochstetteri
Lyciasalamandra_atifi
Paramesotriton_hongkongensis
Pachytriton_feii
Triturus_carnifex
Triturus_macedonicus
Triturus_cristatus
Triturus_karelinii
Triturus_dobrogicus
Triturus_marmoratus
Triturus_pygmaeus
Notophthalmus_perstriatus
Echinotriton_andersoni
Tylototriton_verrucosus
Tylototriton_shanjing
Tylototriton_kweichowensis
Tylototriton_taliangensis
Tylototriton_wenxianensis
Ambystoma_mexicanum
Ambystoma_andersoni
Ambystoma_dumerilii
Ambystoma_tigrinum
Ambystoma_californiense
Ambystoma_laterale
Ambystoma_barbouri
Ambystoma_unisexual
Ambystoma_texanum
Ambystoma_bishopi
Stereochilus_marginatus
Pseudotriton_ruber
Gyrinophilus_porphyriticus
Gyrinophilus_palleucus
Eurycea_bislineata
Oedipina_poelzi
Bolitoglossa_n._sp.
Thorius_n._sp.
Batrachoseps_wrightorum
Batrachoseps_attenuatus
Batrachoseps_nigriventris
Hemidactylium_scutatum
Aneides_flavipunctatus
Aneides_hardii
Ensatina_eschscholtzii
Hydromantes_brunus
Desmognathus_wrighti
Desmognathus_fuscus
Phaeognathus_hubrichti
Plethodon_elongatus
Rhyacotriton_variegatus
Necturus_beyeri
Proteus_anguinus
Ranodon_sibiricus
Batrachuperus_mustersi
Batrachuperus_gorganensis
Hynobius_amjiensis
Hynobius_yiwuensis
Hynobius_chinensis
Hynobius_guabangshanensis
Hynobius_maoershanensis
Hynobius_leechii
Hynobius_yangi
Hynobius_quelpaertensis
Hynobius_nebulosus
Hynobius_nigrescens
Hynobius_formosanus
Hynobius_arisanensis
Hynobius_kimurae
Batrachuperus_londongensis
Batrachuperus_pinchonii
Batrachuperus_tibetanus
Batrachuperus_yenyuanensis
Liua_shihi
Liua_tsinpaensis
Pseudohynobius_puxiongensis
Pseudohynobius_flavomaculatus
Pseudohynobius_jinfo
Pseudohynobius_shuichengensis
Salamandrella_keyserlingii
Salamandrella_tridactyla
Pachyhynobius_shangchengensis
Onychodactylus_fischeri
Onychodactylus_zhaoermii
Onychodactylus_zhangyapingi
Andrias_davidianus
Andrias_japonicus
Typhlonectes_natans
Potomotyphlus_kaupii
Chthonerpeton_indistinctum
Caecilia_volcani
Caecilia_tentaculata
Caecilia_gracilis
Oscaecilia_ochrocephala
Gegeneophis_ramaswamii
Indotyphlus_maharashtraensis
Hypogeophis_rostratus
Grandisonia_sechellensis
Praslinia_cooperi
Dermophis_mexicanus
Gymnopis_multiplicata
Schistometopum_gregorii
Geotrypetes_seraphini
Siphonops_annulatus
Luetkenotyphlus_brasiliensis
Microcaecilia_sp.
Microcaecilia_dermatophaga
Microcaecilia_unicolor
Herpele_squalostoma
Boulengerula_boulengeri
Boulengerula_taitana
Chikila_fulleri
Scolecomorphus_vittatus
Crotaphatrema_lamottei
Ichthyophis_glutinosus
Ichthyophis_bannanicus
Uraeotyphlus_cf.
Uraeotyphlus_gansi
Ichthyophis_bombayensis
Rhinatrema_bivittatum

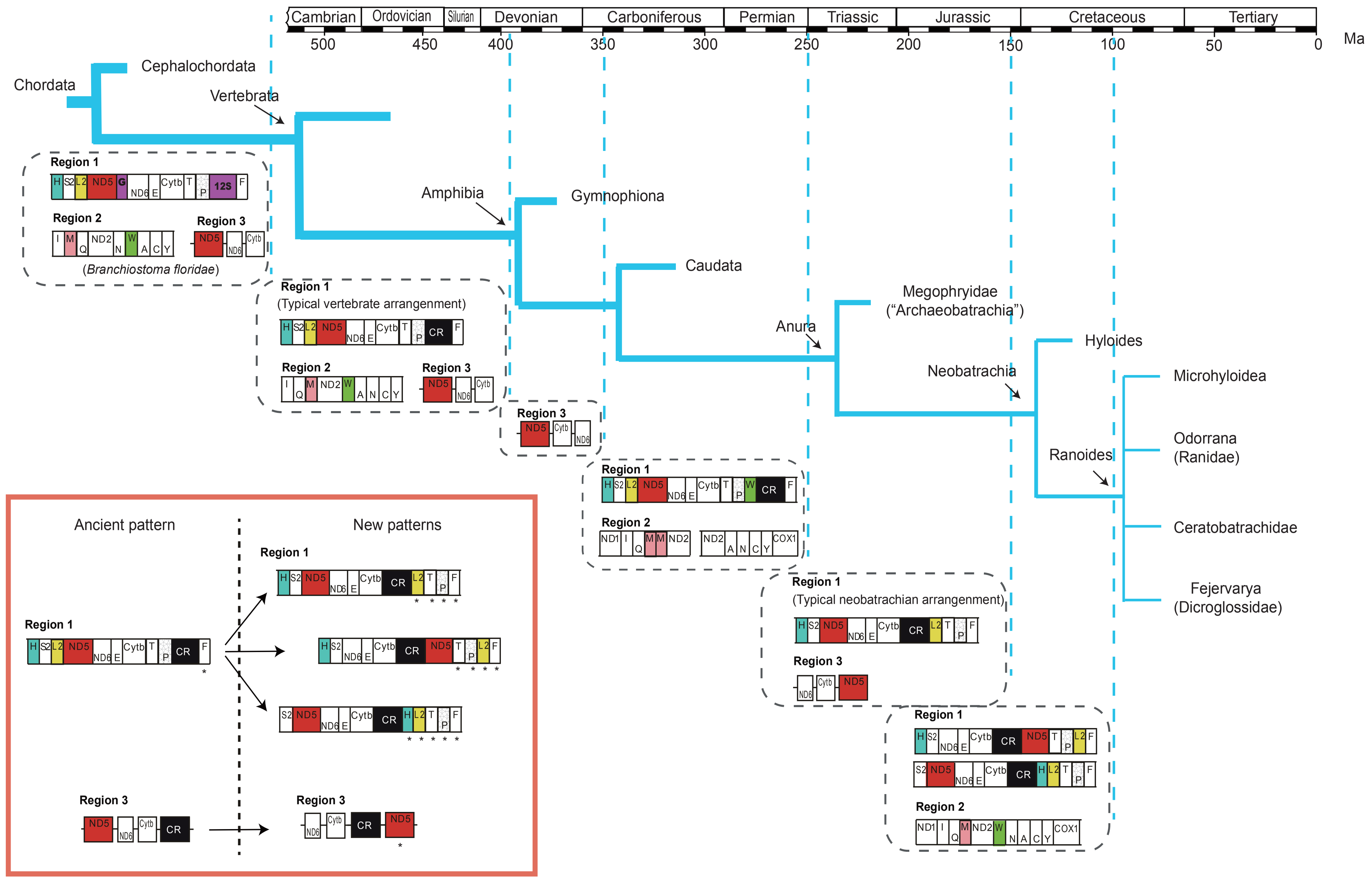

Cambrian
Ordovician
Silurian
Devonian
Carboniferous
Permian
Triassic
Jurassic
Cretaceous
Tertiary
500
450
400
350
300
250
200
150
100
50
0
Ma
Chordata
Cephalochordata
Vertebrata
Amphibia
Gymnophiona
Caudata
Anura
Megophryidae
("Archaeobatrachia")
Neobatrachia
Hyloides
Ranoides
Microhyloidea
Odorrana
(Ranidae)
Ceratobatrachidae
Fejervarya
(Dicroglossidae)
Region 1
Region 2
Region 3
(Branchiostoma floridae)
(Typical vertebrate arrangement)
(Typical neobatrachian arrangement)
Ancient pattern
New patterns

Additional file 5: Word S1

Amplification Scheme and Primers Sequences

We first amplified four large fragments of the mitochondrial genome (that is, *nad4-nad5-nad6-cob, rrnL-nad1-nad2-cox1, cox1-cox2-a8-a6-cox3-nd3-nd4 and CR*) based on the following primers (Table 1) and sequenced them. And further amplification of the remaining fragments was carried out based on the above known sequence and the primer sequences of Reference [1].

Table1 Primer locations and sequences

No.	Upstream primer (5'end)	Downstream primer (3'end)	Target segment
1	CCACCGCTCAAGGGTTTTCTATCG	CTGAGAGGAGGTTGGTGATAACTGTAGC	*nad4-nad5-nad6-cob*
2	GTATCAACGGCATCACGAGGGTTACACT	CTGGGTGACCAAAGAATCAGAAGAG	*rrnL-nad1-nad2-cox1*
3	TGGCACCGCCCATATTATGATTACTTTA	CCCTGACTAAATGCCTTTGCCATCCACT	CR
4	CTCTTCCAGTTTTAGCCGCAGGCATTAC	GAGGGTGGTGAGTTGAAGAAATGTGCTG	*cox1-cox2-a8-a6-cox3-nd3-nd4*

Reference

[1] Zhang J F, Nie L W, Wang Y, et al. The complete mitochondrial genome of the large-headed frog, *Limnonectes bannaensis* (Amphibia: Anura), and a novel gene organization in the vertebrate mtDNA[J]. Gene, 2009, 442(1-2): 119-127.

Additional file 6: Table S2. Determination of substitution saturation for each codon position of each gene.

ATP6 codon I					
NumOTU	Iss	Iss. cSym	*P*	Iss. cAsym	*P*
4	0.367	0.777	0	0.763	0
8	0.369	0.733	0	0.632	0
16	0.373	0.652	0	0.458	0.0475
32	0.381	0.686	0	0.366	0.7252
ATP6 codon II					
NumOTU	Iss	Iss. cSym	*P*	Iss. cAsym	*P*
4	0.169	0.777	0	0.763	0
8	0.17	0.733	0	0.632	0
16	0.173	0.652	0	0.458	0
32	0.177	0.686	0	0.366	0
ATP6 codon III					
NumOTU	Iss	Iss. cSym	*P*	Iss. cAsym	*P*
4	0.816	0.777	0.2486	0.763	0.1189
8	0.824	0.733	0.0042	0.632	0
16	0.821	0.652	0	0.458	0
32	0.826	0.686	0	0.366	0
ATP8 codon I					
NumOTU	Iss	Iss. cSym	*P*	Iss. cAsym	*P*
4	0.559	0.924	0	1.063	0
8	0.578	1.005	0	1.053	0
16	0.586	0.56	0.7705	0.662	0.3943

32	0.584	1.156	0	1.195	0
ATP8 codon II					
NumOTU	Iss	Iss. cSym	*P*	Iss. cAsym	*P*
4	0.383	0.924	0	1.063	0
8	0.411	1.005	0	1.053	0
16	0.41	0.56	0.0831	0.662	0.0044
32	0.415	1.156	0	1.195	0
ATP8 codon III					
NumOTU	Iss	Iss. cSym	*P*	Iss. cAsym	*P*
4	0.758	0.924	0.0355	1.063	0.0002
8	0.754	1.005	0.0021	1.053	0.0003
16	0.767	0.56	0.0082	0.662	0.1712
32	0.765	1.156	0	1.195	0
COX1 codon I					
NumOTU	Iss	Iss. cSym	*P*	Iss. cAsym	*P*
4	0.139	0.796	0	0.762	0
8	0.133	0.753	0	0.641	0
16	0.136	0.722	0	0.513	0
32	0.136	0.703	0	0.378	0
COX1 codon II					
NumOTU	Iss	Iss. cSym	*P*	Iss. cAsym	*P*
4	0.038	0.796	0	0.762	0
8	0.037	0.753	0	0.641	0
16	0.038	0.722	0	0.513	0
32	0.04	0.703	0	0.378	0

COX1 codon III					
NumOTU	Iss	Iss. cSym	*P*	Iss. cAsym	*P*
4	0.741	0.796	0.0133	0.762	0.3339
8	0.739	0.753	0.5109	0.641	0
16	0.736	0.722	0.4989	0.513	0
32	0.736	0.703	0.1088	0.378	0
COX2 codon I					
NumOTU	Iss	Iss. cSym	*P*	Iss. cAsym	*P*
4	0.246	0.777	0	0.762	0
8	0.244	0.732	0	0.631	0
16	0.239	0.654	0	0.459	0
32	0.246	0.685	0	0.363	0.0018
COX2 codon II					
NumOTU	Iss	Iss. cSym	*P*	Iss. cAsym	*P*
4	0.11	0.777	0	0.762	0
8	0.107	0.732	0	0.631	0
16	0.108	0.654	0	0.459	0
32	0.113	0.685	0	0.363	0
COX2 codon III					
NumOTU	Iss	Iss. cSym	*P*	Iss. cAsym	*P*
4	0.706	0.777	0.0392	0.762	0.1035
8	0.721	0.732	0.7297	0.631	0.0054
16	0.717	0.654	0.0414	0.459	0
32	0.718	0.685	0.2652	0.363	0
COX3 codon I					

NumOTU	Iss	Iss. cSym	*P*	Iss. cAsym	*P*
4	0.198	0.778	0	0.758	0
8	0.2	0.732	0	0.627	0
16	0.2	0.665	0	0.464	0
32	0.2	0.682	0	0.356	0
COX3 codon II					
NumOTU	Iss	Iss. cSym	*P*	Iss. cAsym	*P*
4	0.083	0.778	0	0.758	0
8	0.08	0.732	0	0.627	0
16	0.081	0.665	0	0.464	0
32	0.083	0.682	0	0.356	0
COX3 codon III					
NumOTU	Iss	Iss. cSym	*P*	Iss. cAsym	*P*
4	0.714	0.732	0.5476	0.627	0.005
8	0.716	0.665	0.0852	0.464	0
16	0.717	0.682	0.2215	0.356	0
32	0.709	0.778	0.0343	0.758	0.1331
CYTB codon I					
NumOTU	Iss	Iss. cSym	*P*	Iss. cAsym	*P*
4	0.254	0.787	0	0.756	0
8	0.252	0.74	0	0.629	0
16	0.255	0.697	0	0.488	0
32	0.255	0.689	0	0.359	0.0011
CYTB codon II					
NumOTU	Iss	Iss. cSym	*P*	Iss.	*P*

				cAsym	
4	0.097	0.787	0	0.756	0
8	0.098	0.74	0	0.629	0
16	0.098	0.697	0	0.488	0
32	0.099	0.689	0	0.359	0
CYTB codon III					
NumOTU	Iss	Iss. cSym	*P*	Iss. cAsym	*P*
4	0.766	0.787	0.4255	0.756	0.6943
8	0.759	0.74	0.4252	0.629	0
16	0.76	0.697	0.0056	0.488	0
32	0.765	0.689	0.0005	0.359	0
ND1 codon I					
NumOTU	Iss	Iss. cSym	*P*	Iss. cAsym	*P*
4	0.302	0.781	0	0.756	0
8	0.309	0.735	0	0.626	0
16	0.309	0.681	0	0.474	0
32	0.313	0.683	0	0.354	0.2591
ND1 codon II					
NumOTU	Iss	Iss. cSym	*P*	Iss. cAsym	*P*
4	0.132	0.781	0	0.756	0
8	0.13	0.735	0	0.626	0
16	0.13	0.681	0	0.474	0
32	0.131	0.683	0	0.354	0
ND1 codon III					
NumOTU	Iss	Iss. cSym	*P*	Iss. cAsym	*P*

4	0.774	0.781	0.7862	0.756	0.535
8	0.784	0.735	0.0641	0.626	0
16	0.787	0.681	0	0.474	0
32	0.789	0.683	0	0.354	0
ND2 codon I					
NumOTU	Iss	Iss. cSym	*P*	Iss. cAsym	*P*
0.463	0.784	0	0.755	0	0.463
0.464	0.737	0	0.627	0	0.464
0.468	0.688	0	0.481	0.7164	0.468
0.478	0.685	0	0.355	0.0007	0.478
ND2 codon II					
NumOTU	Iss	Iss. cSym	*P*	Iss. cAsym	*P*
4	0.259	0.784	0	0.755	0
8	0.25	0.737	0	0.627	0
16	0.255	0.688	0	0.481	0
32	0.255	0.685	0	0.355	0.0003
ND2 codon III					
NumOTU	Iss	Iss. cSym	*P*	Iss. cAsym	*P*
4	0.821	0.784	0.1785	0.755	0.0186
8	0.836	0.737	0.0001	0.627	0
16	0.833	0.688	0	0.481	0
32	0.838	0.685	0	0.355	0
ND3 codon I					
NumOTU	Iss	Iss. cSym	*P*	Iss.	*P*

				cAsym	
4	0.358	0.797	0	0.829	0
8	0.357	0.781	0	0.719	0
16	0.363	0.595	0.0004	0.473	0.0846
32	0.367	0.776	0	0.535	0.0102
ND3 codon II					
NumOTU	Iss	Iss. cSym	*P*	Iss. cAsym	*P*
4	0.176	0.797	0	0.829	0
8	0.176	0.781	0	0.719	0
16	0.17	0.595	0	0.473	0
32	0.176	0.776	0	0.535	0
ND3 codon III					
NumOTU	Iss	Iss. cSym	*P*	Iss. cAsym	*P*
4	0.79	0.797	0.8871	0.829	0.419
8	0.803	0.781	0.6168	0.719	0.0657
16	0.798	0.595	0	0.473	0
32	0.798	0.776	0.5989	0.535	0
ND4L codon I					
NumOTU	Iss	Iss. cSym	*P*	Iss. cAsym	*P*
4	0.465	0.792	0	0.759	0
8	0.47	0.747	0	0.636	0
16	0.465	0.713	0	0.503	0.2157
32	0.472	0.697	0	0.37	0.0014
ND4L codon II					
NumOTU	Iss	Iss. cSym	*P*	Iss. cAsym	*P*

4	0.277	0.792	0	0.759	0
8	0.266	0.747	0	0.636	0
16	0.267	0.713	0	0.503	0
32	0.271	0.697	0	0.37	0.0002
ND4L codon III					
NumOTU	Iss	Iss. cSym	*P*	Iss. cAsym	*P*
4	0.835	0.792	0.0679	0.759	0.0011
8	0.847	0.747	0	0.636	0
16	0.842	0.713	0	0.503	0
32	0.847	0.697	0	0.37	0
ND4 codon I					
NumOTU	Iss	Iss. cSym	*P*	Iss. cAsym	*P*
4	0.464	0.813	0	0.863	0
8	0.447	0.811	0	0.766	0
16	0.45	0.583	0.043	0.495	0.4853
32	0.462	0.828	0	0.628	0.0127
ND4 codon II					
NumOTU	Iss	Iss. cSym	*P*	Iss. cAsym	*P*
4	0.472	0.779	0	0.757	0
8	0.476	0.733	0	0.626	0.0001
16	0.479	0.671	0	0.468	0.7748
32	0.485	0.682	0	0.354	0.0013
ND4 codon III					
NumOTU	Iss	Iss. cSym	*P*	Iss. cAsym	*P*
4	0.805	0.813	0.8902	0.863	0.2718

8	0.815	0.811	0.9247	0.766	0.3044
16	0.811	0.583	0	0.495	0
32	0.814	0.828	0.7285	0.628	0
ND5 codon I					
NumOTU	Iss	Iss. cSym	*P*	Iss. cAsym	*P*
0.429	0.8	0	0.768	0	0.429
0.421	0.759	0	0.648	0	0.421
0.428	0.734	0	0.523	0.0005	0.428
0.434	0.71	0	0.382	0.0613	0.434
ND5 codon II					
NumOTU	Iss	Iss. cSym	*P*	Iss. cAsym	*P*
4	0.26	0.8	0	0.768	0
8	0.258	0.759	0	0.648	0
16	0.259	0.734	0	0.523	0
32	0.263	0.71	0	0.382	0
ND5 codon III					
NumOTU	Iss	Iss. cSym	*P*	Iss. cAsym	*P*
4	0.814	0.8	0.5021	0.768	0.0255
8	0.813	0.759	0.0031	0.648	0
16	0.818	0.734	0	0.523	0
32	0.818	0.71	0	0.382	0
ND6 codon I					
NumOTU	Iss	Iss. cSym	*P*	Iss. cAsym	*P*

4	0.481	0.779	0	0.783	0
8	0.497	0.744	0	0.657	0.0018
16	0.499	0.624	0.0188	0.454	0.3884
32	0.512	0.71	0.0002	0.415	0.0692
ND6 codon II					
NumOTU	Iss	Iss. cSym	*P*	Iss. cAsym	*P*
4	0.311	0.779	0	0.783	0
8	0.307	0.744	0	0.657	0
16	0.313	0.624	0	0.454	0.0031
32	0.321	0.71	0	0.415	0.053
ND6 codon III					
NumOTU	Iss	Iss. cSym	*P*	Iss. cAsym	*P*
4	0.825	0.779	0.2555	0.783	0.298
8	0.842	0.744	0.0078	0.657	0
16	0.854	0.624	0	0.454	0
32	0.853	0.71	0	0.415	0

OUT, operational taxonomic units; Iss, index of substitution saturation; Iss. cSym, Iss.c assuming a symmetrical topology; Iss.cAsym, Iss.c assuming an asymmetrical topology; Iss.c, critical index of substitution saturation.

Additional file 7: Table S3. The best partitioning scheme selected by PartitionFinder for different data matrices.

Optimal Partition	Model	Initial Partition
Partition 1	GTR+I+G	CYTB(codon1), ND4L(codon1), ND3(codon1), ATP6(codon1), ND1(codon1), ND5(codon1), ND4(codon1), ND2(codon1), ATP8(codon2), ATP8(codon1), ND6(codon2), ND6(codon1), COX1(codon1), COX3(codon1), COX2(codon1)
Partition 2	GTR+I+G	COX1(codon2), CYTB(codon2), COX3(codon2), ND1(codon2), COX2(codon2), ATP6(codon2), ND5(codon2), ND2(codon2), ND4(codon2), ND3(codon2), ND4L(codon2)
